# Perinatal Programming of Circadian Clock-Stress Crosstalk

**DOI:** 10.1155/2018/5689165

**Published:** 2018-02-08

**Authors:** Mariana Astiz, Henrik Oster

**Affiliations:** Institute of Neurobiology, Center of Brain, Behavior & Metabolism, University of Lübeck, Marie-Curie Street, 23562 Lübeck, Germany

## Abstract

An intact communication between circadian clocks and the stress system is important for maintaining physiological homeostasis under resting conditions and in response to external stimuli. There is accumulating evidence for a reciprocal interaction between both—from the systemic to the molecular level. Disruption of this interaction by external factors such as shiftwork, jetlag, or chronic stress increases the risk of developing metabolic, immune, or mood disorders. From experiments in rodents, we know that both systems maturate during the perinatal period. During that time, exogenous factors such as stress or alterations in the external photoperiod may critically affect—or *program*—physiological functions later in life. This developmental programming process has been attributed to maternal stress signals reaching the embryo, which lastingly change gene expression through the induction of epigenetic mechanisms. Despite the well-known function of the adult circadian system in temporal coordination of physiology and behavior, the role of maternal and embryonic circadian clocks during pregnancy and postnatal development is still poorly defined. A better understanding of the circadian-stress crosstalk at different periods of development may help to improve stress resistance and devise preventive and therapeutic strategies against chronic stress-associated disorders.

## 1. Introduction: Regulation of Glucocorticoid Release

**Figure 1:**
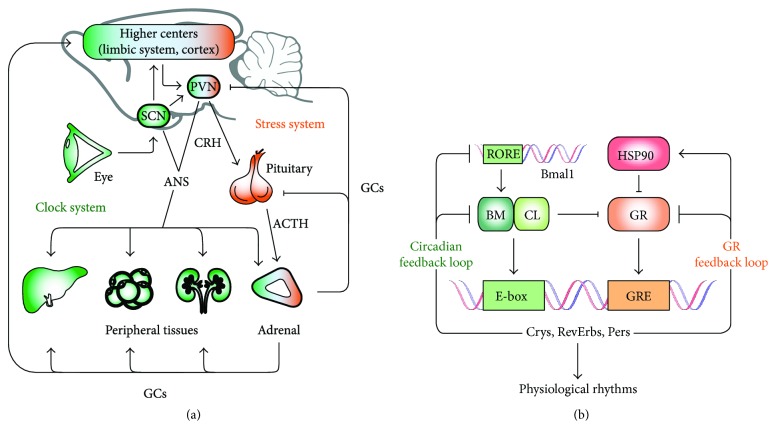
Clock-stress coupling at systemic and molecular levels. (a) The circadian clock and stress systems influence each other's activity at multiple and reciprocal levels. The central clock in the suprachiasmatic nucleus (SCN) of the hypothalamus is under the regulation of the light input from the retina. SCN controls the circadian function of the hypothalamus-pituitary-adrenal (HPA) axis to induce a rhythmic production and secretion of glucocorticoid (GCs) hormones from the adrenal glands. Via autonomic nervous system (ANS) pathways, the SCN further synchronizes adrenal clocks to regulate the sensitivity of the steroidogenic machinery to adrenocorticotropic hormone (ACTH) stimulation. Peripheral clocks in liver, adipose tissue, and kidney are regulated by the SCN through the ANS and rhythmic entraining signals such as GCs. During acute stress, brainstem and limbic forebrain nuclei activate the HPA axis through the paraventricular nucleus (PVN) of the hypothalamus, resulting in the acute production of GCs by the adrenal cortex. About one hour after acute stress stimulation, GC levels return to baseline due to the activation of a negative feedback mechanism. GCs inhibit the synthesis of corticotropin-releasing hormone (CRH) in the PVN and ACTH in the pituitary, downregulating the stress system activity and shutting down steroid production at the level of the adrenal cortex. (b) The coupling between the circadian clock and the stress system relays, at molecular level, on two parallel transcriptional-translational feedback loops (TTLs) that modulate each other. Hormone-bound GR binds glucocorticoid responsive elements (GREs) in the promoter region of several clock genes and various clock-controlled genes. Conversely, CLOCK (CL)/BMAL1 (BM) heterodimers (active during the night) interact physically and acetylate GR, thereby reducing its affinity to GREs and its translocation into the nucleus. CRY1 and CRY2 can interact with the C-terminal domain of GR in a ligand-dependent fashion, repressing the GR-mediated transactivation of certain target genes. Additionally, REV-ERB*α* (active during the day as an inhibitor of BMAL1 expression) can stabilize the nuclear localization of GR reinforcing its transcriptional activity, through its interaction with heat shock protein 90 (HSP90). Several genes contain both, GRE and E-box elements in the promoters being regulated by both loops. Through this complex network of interactions, GR and the clock machinery finally translate environmental information in physiological responses.

In most animal species, an internal 24-hour timing system known as *circadian clock* coordinates behavioral and physiological processes to adapt to daily recurring changes in the environment [1]. The mammalian circadian system is organized in a hierarchical way with a master pacemaker located in the hypothalamic suprachiasmatic nucleus (SCN) and subordinated clocks found throughout the brain and periphery [2]. The SCN perceives time of day via direct photic input from the retina and subsequently relays temporal information to the body [3, 4]. Peripheral clocks are able to measure time even in the absence of the SCN [5]. However, temporal resetting signals (zeitgebers) from the SCN are required to synchronize the different peripheral oscillators with each other and with the external time *in vivo* [3, 6, 7]. The mechanism of this systemic circadian entrainment is still poorly understood. So far, we know that the SCN uses both humoral and neuronal pathways to transmit time information to peripheral clocks [1, 8]. Among the most studied mediators of circadian entrainment are glucocorticoids (GCs) that also play an essential role, together with catecholamines, in response to stress [9]. Under nonstressed conditions, circulating GC levels display strong daily rhythmicity peaking at the beginning of the active phase (i.e. the morning in humans and the evening in nocturnal rodents). These circadian GC rhythms are implicated in the coordination of clock function in central and peripheral tissues [10, 11] Figure 1(a).

The circadian control of GC secretion results from a cooperation of the SCN pacemaker and tissue clocks along the hypothalamus-pituitary-adrenal (HPA) axis [3]. The SCN controls the rhythmic secretion of adrenocorticotropic hormone (ACTH) from the pituitary, via the regulation of corticotropin-releasing hormone (CRH) and arginine vasopressin (AVP) release from the paraventricular nucleus of the hypothalamus (PVN). ACTH, in turn, stimulates GC production in the *zona fasciculata* of the adrenal cortex Figure 1(a). Via autonomic pathways, the SCN also synchronizes adrenal clocks, regulating the time-of-day-dependent sensitivity of the steroidogenic machinery to ACTH stimulation [3, 12–14]. Thus, an intact circadian clock network along the HPA axis is required for a robust rhythmic secretion of GCs [3, 14].

Besides this, during stress, brainstem and limbic forebrain nuclei activate the HPA axis through the PVN, resulting also in the release of GCs from the adrenal cortex [15]. About one hour after acute stress stimulation, GC levels return to baseline due to the activation of a negative feedback mechanism [16]. By binding to glucocorticoid (GR) and mineralocorticoid receptors (MR), GCs inhibit the synthesis of CRH in the hypothalamic PVN and ACTH in the pituitary, downregulating the stress system and shutting down steroid production at the level of the adrenal cortex [17], Figure 1(a).

## 2. Pathological Consequences of GC Rhythm Disruption

By acting as an entrainment signal for circadian clocks throughout the body, GC rhythms play a key role in coordinating carbohydrate, lipid, and protein metabolism. For example, it was shown that in the liver, many genes involved in carbohydrate metabolism exhibit diurnal expression rhythms. For some of these genes, the rhythmic regulation depends on local hepatocyte clock function, but others are under direct GC control [18–21]. Regarding behavior, circadian GC secretion is essential in the regulation of sleep, mood, and cognition. Animal studies show that GCs are able to influence rhythmic brain functions by entraining central clocks as well as by interacting with neuromodulatory pathways such as the serotonergic system [22, 23].

Therefore, disruption of circadian GC rhythms can have numerous pathological outcomes. Various lifestyle-associated factors such as shiftwork, social stress, sleep disruption, mistimed eating, or jetlag can alter GC rhythmicity and thereby disrupt downstream physiology [24]. For example, extended shiftwork is associated with metabolic disorders such as obesity, cardiovascular diseases, insulin resistance, and hyperlipidemia [25], while repeated jetlag and sleep deprivation may lead to mood disorders and cognitive impairments [26, 27].

## 3. Cellular Mechanism of Clock-Stress Crosstalk

A coupling between the stress system and the circadian clock occurs not only at systemic but also at molecular level [28] Figures 1(a) and 1(b). At target cells, GCs bind and activate two intracellular receptors, MR and GR. Due to its high affinity for GCs, MR is constitutively activated under most physiological conditions. GRs, in contrast, are only activated by higher GC concentrations, conveying phasic responses, for example, at the circadian peak or during acute stress situations [29]. GC-GR signaling is essential to maintain physiological homeostasis in response to external stimuli and has a key function for the coupling between the circadian and stress systems [30, 31]. GRs act as ligand-activated transcription factors. Upon GC binding, GC-GR dissociates from heat shock factors (such as HSP90) and translocates from the cytosol into the nucleus, where they bind to glucocorticoid responsive element *(GRE)* DNA motifs in regulatory regions of target genes to modulate transcription [31] (Figure 1(b)).

The cellular circadian clockwork present in almost all cells in the body is based on a set of *clock genes* organized in a system of interlocked transcriptional-translational feedback loops (TTLs). Time-of-day information is translated from the clock machinery into physiological signals through rhythmic regulation of downstream clock-controlled genes [32]. In nocturnal animals, the transcription factors CLOCK (circadian locomotor output cycles kaput) and BMAL1 (brain and muscle aryl hydrocarbon receptor nuclear translocator-like 1; official symbol: ARNTL) bind to *E-box* promoter elements during the night, to drive the expression of three *Per* (period 1–3) and two *Cry* (cryptochrome 1/2) genes. PER and CRY proteins form complexes in the cytoplasm that—during the day—translocate into the nucleus to inhibit CLOCK/BMAL1 activity, shutting down their own transcription. After degradation of nuclear PER/CRY complexes, the inhibition of CLOCK/BMAL1 is released and a new circadian cycle begins [33], Figure 1(b).

GR signaling and the molecular clock machinery interact in multiple and reciprocal ways. Hormone-bound GR binds *GREs* in the promoter regions of several clock genes such as *Per2* [32, 34]. The nuclear receptor *Rev-ERBα*, which represses the transcription of *Bmal1*, contains negative *GREs* mediating GR transrepression [35]. The clock gene *Per1* contains both, *GR* and *E-box* elements in its regulatory sequences [36]. The presence of both, *GRE* and *E-boxes*, has also been reported for other genes that are not part of the core circadian TTL [21]. Besides transcriptional regulation, recent studies suggest that clock proteins and GR can interact physically. CLOCK is able to acetylate the hinge region lysine cluster of GR, reducing its DNA-binding [37]. CRY proteins directly bind GR, thereby decreasing its transactivation potential [38]. Finally, the presence of REV-ERB*α* influences the stability and nuclear localization of GR through its interaction with heat shock protein 90 (HSP90) [39] (Figure 1(b)).

In the adrenal glands and in some non-SCN brain regions, GR and clock genes further interact in modulating catecholamine biosynthesis and degradation, thus reinforcing the coupling between circadian and stress systems (reviewed in [40]). Transcription of monoamine oxidase I (*Maoa*), whose product is involved in catecholamine degradation, is directly activated by CLOCK/BMAL1 [41]. At the same time, catecholamine biosynthesis is also clock regulated and the transcription of one of its pacemaker enzymes, tyrosine hydroxylase *(TH)*, is repressed by *Rev-ERBα* [42]. A direct link between circadian GC and catecholamine synthesis is established by GRs activating the nuclear orphan receptor NURR1 (NR4A2) to induce the expression of *TH*, thereby promoting catecholamine production [43]. GR also regulates the expression of catechol-O-methyltransferase (*Comt*) involved in catecholamine catabolism. In summary, a complex network of interactions between GR and the clock machinery controls time-of-day-dependent stress responses through regulation of GR transcriptional activity and catecholamine metabolism [44].

## 4. The Impact of Stress on Circadian Entrainment

The rise in GC blood levels right before the active phase allows to anticipate periods of higher energy demands and increased probability of encountering stressful situations [15]. Many of the processes involved in this anticipation are under circadian clock control and are supported by the strong entrainment effects of GCs on different peripheral and central circadian oscillators [10]. In a transgenic rat model expressing a luciferase reporter under the control of *Per1* promoter, adrenalectomy shifts the clock only in some, but not all tissues. This indicates that GC entrainment effects are highly tissue specific [45]. In addition to their effect on clock gene expression, GCs can entrain locomotor activity and—at least in mice—a manipulation of the phase of GC circadian release can accelerate behavioral adaptation under jetlag conditions [46].

In acute or chronic stressful situations, increased GC release may reset the phase of the circadian clock system (reviewed in [47]). In a recent paper, Tahara and colleagues showed that restraint stress in mice-induced differential changes in the phase and amplitude of *Per2* expression in peripheral tissues (kidney, liver, and submandibular gland) depending on the time of the day. A stress challenge applied at the beginning of the light phase induced a phase advance, while stress at the beginning of the dark phase caused phase delays of *Per2* expression [48].

## 5. Temporal Regulation of HPA Axis Responsiveness

In parallel, the extent of stress responses is dependent on the time of the day and on the nature of the stressor [49–52]. During the inactive phase, restraint/immobilization, foot shock, or shaking stress results in a stronger increase in GC and ACTH release than during the active phase [53, 54]. There is also evidence of a time-dependent adaptive response to repeated and predictable stress exposure [55]. Moreover, genetic disruption in the circadian system dramatically alters stress system's activity. Interestingly, the impact of clock gene deletion on circulating GCs depends on which member of the TTL is missing. Mice lacking a gene from the positive limb of the molecular circadian system, such as BMAL1 or CLOCK, show hypocortisolism and insensitivity to acute stress in terms of behavioral and hormonal response [56, 57]. On the other hand, mice lacking genes of the negative limb of the TTL have shown both hyper- and hypocortisolism [58].

## 6. Perinatal Development and Programming of the Circadian Stress System in Rodents

As outlined above, numerous studies in animals indicate that adaptation to the environment is achieved by the coupling between the circadian and stress systems through a highly conserved and interrelated regulatory network. Interestingly, in mammals, this network is built during a critical period of perinatal life. During this time, adverse environmental conditions interact with the genetic background to *program* the coupling and, thereby, the responses to the environment later in life. Several theoretical models have been proposed to explain the long-term effects of early adversity, since, depending on the circumstances, it can result in either vulnerability or resilience to later experiences (reviewed in [59]). Such perinatal programming process has been attributed to maternal signals (e.g., glucocorticoids, catecholamines, melatonin, and dopamine) reaching the embryo or the newborn, lastingly changing gene expression through the induction of epigenetic mechanisms [60].

## 7. Long-Term Outcomes of Stress or Circadian Disruption during Development

Interestingly, both circadian disruption and stress during pregnancy program adult metabolism and behavior similarly [61–64]. Mice exposed to constant light either during the prenatal or perinatal period show reduce growth rates, impair emotion behavior and energetic metabolism, elevate cognitive deficits and fear responses in the long-term [65–68]. Pregnant rats exposed to repeated photoperiod shifts showed altered circadian rhythms (activity, temperature, food consumption, heart rate, and hormone profiles). Their offspring showed impaired carbohydrate metabolism, increased adiposity, altered sensitivity to leptin and insulin, and impaired responses to stress in adulthood [64, 69]. In a recent paper, Smarr and colleagues [70] showed that the outcomes of chronic maternal circadian disruption (consisting of 6 h advances in the light cycle every 4 days) are not prevented by cross-fostering with undisturbed mothers, highlighting the importance of the prenatal period for programming the adult phenotype through circadian disruption. However, the early postnatal light environment alters maternal care behavior by disrupting activity rhythms or by inducing stress and seems to impact on the offspring's development as well [71–73]. Exposure to constant light conditions during the suckling stage in mice programs mRNA expression of *CRH* in the PVN later in life [66]. In rodents, a high concentration of CRH in the PVN is associated with increased despair behavior [67, 74].

A widely used protocol for inducing prenatal stress consists on restraining the movement of pregnant rats by confining them to a transparent cylinder, three times a day for 45 min, during the second half of gestation (*prenatal restrain stress*—PRS) [75]. Adult offspring of these mothers show prolonged corticosterone production after acute stress and reduced expression of GR in the hippocampus [76]. HPA axis hyperactivity is observed in PRS rats, accompanied by enhanced sensitivity to drug abuse [77], learning impairments in aged animals [78], altered emotion behaviors related to anxiety and depression [79], and changes in sleep patterns [80, 81]. Other interventions during pregnancy such as prenatal hypoxia lead to altered circadian patterns of activity in standard (12 h : 12 h) light-dark conditions and exaggerated responses to acute stress [82]. In humans, alcohol abuse during pregnancy is deleterious for the normal development of the fetal brain, affecting sleep-wake regulation as well as stress responsiveness [83].

Several experimental models have been used to study the importance of the postnatal period in the programming process. Using maternal separation and cross-fostering experiments has demonstrated that the mother-newborn relationship is important for the development of the stress system [84]. Maternal separation alters peripheral levels of GC, decreases expression of GR in the hippocampus in mice [84], and leads to exaggerated stress and fear responses [85]. However, recently, Santarelli et al. [86] demonstrated that other early postnatal interventions actually confer resilience against chronic stress in adulthood. Variations in the degree of stress generated by maternal separation may be the reason for these apparently conflicting results.

## 8. Development of Stress and Circadian Clock Systems

**Figure 2:**
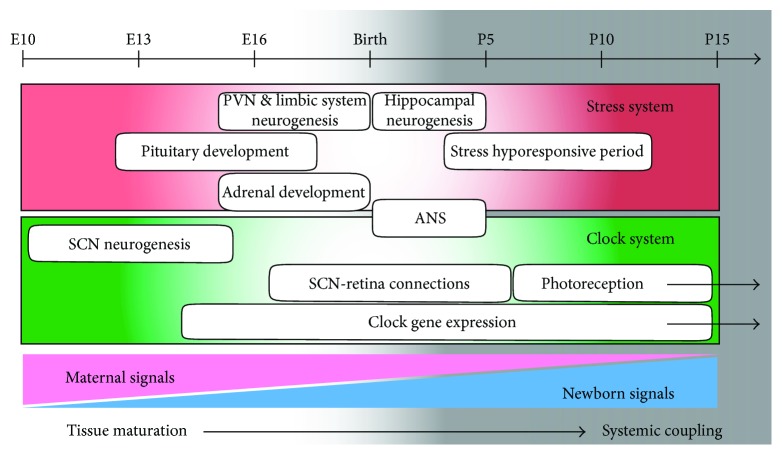
Schematic developmental timeline of coupling in mice. For the rodent circadian clock and stress system development, both, pre- and postnatal periods are critical. During the second half of gestation, the embryonic PVN and limbic system (LS) undergo active neuronal division and intense synaptic organization. The pituitary starts developing earlier, independently from hypothalamic connections. The development of the steroidogenic function of the adrenal cortex also occurs during this period, depending on the secretion of ACTH. The innervation of the adrenal medulla by sympathetic preganglionic nerves occurs soon before birth. The second important period takes place immediately after birth. The hippocampal neurogenesis in rodents is followed by a stress hyporesponsive period (SHRP), after which the HPA axis consolidates and responds in an adult-like way. The development of circadian rhythmicity in rodents occurs in similar periods. In mice, neuronal division in the developing SCN takes place between embryonic day (E)10–15 peaking at E12. Intra-SCN circuits differentiate during the following days and retinal projections reach the SCN shortly after birth. In contrast, the molecular clock machinery in the SCN and peripheral tissues is expressed earlier. From left to right, we represent the embryo development at tissue level (predominantly driven by maternal signals), followed by the development of the systemic coupling for which the newborn signals become essential.

It is interesting to note that, despite the different nature of these interventions, the perinatal period represents a critical time window in which the coupling between the circadian and stress system can be programmed by the environment, Figure 2. For the rodent HPA axis, at least two developmental periods have been identified as critical for shaping its function later in life (reviewed in [87]). The first takes place during the second half of gestation. During this time, the embryonic PVN and limbic system structures undergo active neuronal division and intense synaptic reorganization [88]. Meanwhile, the pituitary develops independently from hypothalamic connections, because the expression of POMC (proopiomelanocortin) and POMC-derived peptides in the pituitary is observed prior to the onset of CRH expression in the PVN [89]. The development of the steroidogenic function in the adrenal cortex occurs later, depending on the pituitary ACTH secretion [90]. The innervation of the adrenal medulla by sympathetic preganglionic nerves occurs soon before birth; during this process, baseline levels of GCs are necessary to induce catecholamine synthesis [91].

The second important period follows immediately after birth. The development of the stress system in rodents is characterized by a stress hyporesponsive period (SHRP) between postnatal day (P) 4 and 14 in rats and slightly earlier in mice [92, 93]. This period is characterized by reduced corticosterone responses to ACTH and various stressors and seems to be strongly dependent on maternal-newborn interaction [94]. In fact, maternal nursing behavior is critical to maintain adrenal hyporesponsiveness [95]. During this period, the hippocampus, which continues maturation, may be the most vulnerable region to the effects of stress ([96, 97]). After the SHRP, the HPA axis of the offspring consolidates and starts responding in an adult-like way [93]. Of note, some studies propose adolescence as a third critical window in HPA axis maturation [98]. In this period, though, stress effects may be mediated primarily through the frontal cortex. In general, juvenile HPA axis function is characterized by a prolonged activation after stress induction compared to adults, which is attributed to an incomplete maturation of the negative feedback mechanism [99].

At the molecular level, the perinatal development of the HPA axis is regulated by soluble vectors such as growth factors, neuropeptides, and hormones [100]. Thus, it is likely that different environmental conditions transmitted by maternal signals reaching the embryo/newborn affect this process.

The development of circadian rhythmicity in rodents occurs in similar periods as that of the stress system. In mice, neuronal division in the developing SCN takes place between embryonic day (E)10–15 peaking at E12 [101]. Intra-SCN circuits differentiate during the following days, and retinal projections reach the SCN mediating the photic entrainment shortly after birth [102]. In contrast, the molecular clock machinery in the SCN and peripheral tissues is already expressed earlier [103]. During midgestation, SCN explants, as well as isolated neurons, are capable of generating molecular oscillations that gradually gain robustness towards birth [104]. However, it is still under discussion when the full development of metabolic and behavioral rhythms together with the response to systemic zeitgebers occurs [105]. Maternal behavioral rhythms such as locomotor activity, body temperature, and milk availability have been implicated in entrainment of embryonic and newborn clocks [106]. It has been proposed that clocks in the embryo and the newborn act just like peripheral oscillators entrained by rhythmic maternal signals passing through placenta or breast milk [107]. Indeed, in a temporal food restriction experiment, while the maternal SCN clock is phase-locked to the light-dark cycle, the embryonic clocks are entrained by maternal food availability, as a peripheral clock would do [108].

GCs have been widely recognized as developmental keys, inducing or repressing transcripts involved in growth and maturation processes [109]. Sufficient GC levels are essential for normal maturation of the central nervous system and peripheral tissues [110]. Therefore, GR is expressed in most embryonic tissues including the placenta and is essential for survival [111]. Excess or deficient GC signaling during the critical programming windows may alter the developmental trajectory of embryonic or newborn tissues, with permanent consequences [112]. During pregnancy, maternal GC levels show a strong circadian variation, which is not translated to the embryo. Embryonic GC concentrations remain stable across the day due to the presence of an enzymatic barrier in placenta, which inactivates GC [113, 114]. However, in stressful situations, high concentrations of GCs can saturate this barrier and reach embryonic tissues, interfering with developmental programs of the circadian clock, stress system and their coupling [115]. As a result of repeated perinatal stressful interventions, an increased DNA methylation in the *GR* promoter and reduced expression of GR have been shown in the hippocampus [116]. Such epigenetic modifications have been proposed as a possible underlying mechanism for an altered regulation of the HPA axis (reviewed in [117]). GR signaling in the hippocampus inhibits the release of CRH/AVP from the PVN, reinforcing the negative feedback mechanism exerted by GC at the PVN itself [118]. GC feedback and HPA axis function can be improved in prenatally stressed mice by maternal sensory stimulation. By cross-fostering experiments, it has been shown that licking and grooming behavior can reduce GR promoter methylation and increase GR expression in the hippocampus [119]. Besides *GR*, similar epigenetic changes have been reported for other HPA axis regulatory genes [120]. In rodents exposed to postnatal stress paradigms based on maternal separation, *POMC* and *CRH* genes were found hypomethylated in the offspring's pituitary and PVN, respectively [121, 122].

The role of maternal catecholamines has been poorly studied as programming factors (reviewed in [123]). Their hydrophilic nature and the lack of specific transporters limit the concentration of catecholamines in the embryonic blood even in acute stress situations. Therefore, the reported adverse effect of high levels of catecholamines during development has been related to an alteration of the uteroplacental circulation which affects fetal oxygen supply [124].

The circadian hormone melatonin is produced by the maternal pineal gland with a strong rhythmicity peaking during the dark phase. It can cross the placenta unaltered, being considered a strong candidate for transmitting temporal information from the mother to the embryo. Interestingly, melatonin receptor 1 expression is particularly strong in the embryonic SCN [125, 126]. A disruption of melatonin rhythmicity by maternal exposure to constant light changes the expression of clock genes in several embryonic tissues. This effect can be prevented by daily injections of melatonin to the mother [127]. After birth, melatonin is transmitted through milk providing a reliable rhythmic signal to the pups during the breastfeeding period [108]. Besides GCs and xmelatonin, dopamine is also able to entrain fetal clocks through D1 receptors, complementing the nocturnal melatonin signal during the day [128]. Still, the development of the circadian system and the mechanism of clock entrainment during fetal and newborn life requires more investigation. Indeed, to add further complexity, it seems that this process is not fully dependent on maternal rhythmicity, since the circadian system in offspring from mothers lacking a functional clock (due to SCN lesions or genetic clock deletion) develops normally [129–131]. Overall, these results provide strong evidence for a role of the prenatal environment and postnatal maternal behavior as critical programming factors.

## 9. Medical Implications of Clock-Stress Coupling

Interestingly, many of the effects induced by early adverse environments in rodents reviewed above were also found in humans (reviewed in [132]). Children from mothers who reported altered sleep rhythms, stress, anxiety, or depression during pregnancy show a higher incidence of attention deficits, impulsivity, and mood disorders [133]. In addition, long-term impairments of circadian cortisol release and higher basal cortisol levels are found in these children, possibly underlying the impairment on sleep, behavioral, and emotional functions [134–137]. Circadian disruptions such as shiftwork or jetlag are considered as risk factors for abnormal brain development during pregnancy. Several epidemiological studies in women further show an association between shiftwork and increased risk of spontaneous abortion, premature delivery, and low birth weight [138].

It seems clear that avoiding circadian disruptions and/or classical stressors during pregnancy would improve the child's health and quality of life. Circadian disruptions may be minimized avoiding mistimed light exposure, reducing blue light illumination in the evening and increasing bright light exposure in the morning [139]. Additionally, scheduled food consumption and activity during the correct time-of-day would help to keep the maternal clock system aligned [140].

Besides the maternal clock, it is also important to consider that pregnant women at risk of giving birth before term are treated with GCs to accelerate fetal lung development. Several epidemiological studies show that such prenatal GC treatment induces long-term behavioral and metabolic deleterious effects in children [141–143]. Further long-term studies are in progress and will help to improve the dose and time of administration of such treatments [144]. As in rodents, the human HPA axis and the circadian system develop during late prenatal and early postnatal periods [145]. Thus, another aspect that should be considered is the exposure to constant bright light in preterm neonatal care units. Several illumination strategies have recently been compared showing that rhythmic light-dark cycles improve sleep development and weight gain in newborns [146, 147].

To conclude, it becomes increasingly clear that circadian and stress regulation is tightly coupled at all levels of organization. Targeting the circadian-stress crosstalk has high medical potential regarding metabolic and cognitive chronic disorders, both from a preventive and a therapeutic perspective.
